# Preparing students to deal with the consequences of the workforce shortage among health professionals: a qualitative approach

**DOI:** 10.1186/s12909-022-03819-4

**Published:** 2022-11-04

**Authors:** Christoph Golz, Annie Oulevey Bachmann, Tiziana Sala Defilippis, Andrea Kobleder, Karin Anne Peter, René Schaffert, Xenia Schwarzenbach, Thomas Kampel, Sabine Hahn

**Affiliations:** 1 Department of Health Professions, Bern University of Applied Sciences, Murtenstrasse 10, 3008 Bern, Switzerland; 2 La Source School of Nursing, HES-SO University of Applied Sciences and Arts Western Switzerland, Lausanne, Switzerland; 3 Department of Business Economics, Health and Social Care, University of Applied Sciences and Arts of Southern Switzerland, Locarno, Switzerland; 4 School of Health Professions, Zurich University of Applied Sciences, Winterthur, Switzerland; 5 Department of Health, Eastern Switzerland University of Applied Sciences, St. Gallen, Switzerland

**Keywords:** Bachelor’s program, Curriculum, Health professionals, Knowledge mapping, Master’s program, Workforce shortage

## Abstract

**Background:**

Healthcare is facing a shortage of qualified healthcare professionals. The pandemic has brought to light the fragile balance that affects all healthcare systems. Governments have realized that these systems and the professionals working in them need support at different levels to strengthen the retention of the workforce. Health professionals’ education can play an important role in ensuring that new generations of workers have sound personal and professional competencies to successfully face the challenges of professional practice. These challenges are described in the literature, but the extent to which they are considered in health professionals’ education is less clear.

**Methods:**

This qualitative study compares the professional challenges and educational needs described in the literature with the current curricula for health professionals offered in Switzerland. Data were collected nationally through focus group interviews with 65% of Switzerland’s directors of bachelor’s and master’s programs of health professions (nursing, physiotherapy, occupational therapy, midwifery, nutrition and dietetics, osteopathy, radiologic medical imaging technology, health promotion and prevention, and health sciences). The data attained were analyzed using knowledge mapping.

**Results:**

The results reveal a gap among education programs with regard to occupational health promotion and cultural diversity. Both topics are taught with a sole focus on patients, and students are expected to adopt similar strategies for their health promotion and stress management. Physicians are insufficiently involved in interprofessional education. The programs fail to enhance health professionals’ political, economic and digital competencies.

**Conclusion:**

The results of this study offer clear guidance about what topics need to be integrated into curricula to improve health professionals’ well-being at work and their preparedness to face daily professional challenges.

**Supplementary Information:**

The online version contains supplementary material available at 10.1186/s12909-022-03819-4.

## Introduction

Health systems around the globe are in need of new generations of health professionals [1]. Indeed, most countries are facing a substantial workforce shortage among health professionals. By 2030, more than 18 million additional health professionals will be needed worldwide [2]. The reasons for this shortage are twofold. On the one hand, staffing needs are growing due to the increasing health needs of populations in the context of globally aging societies and rises in chronic diseases [1, 3]. On the other hand, health systems are struggling to recruit and retain workers because younger generations widely deem these professions unattractive, and the working conditions experienced by professionals once they have completed their education tend to be difficult [4–7]. Even Switzerland, a high-income country, is affected by a workforce shortage in the healthcare system. In response, the country has invested in the education and training of new health professionals [8]. However, the retention of health professionals also needs investment [2]. Therefore, in 2017 a national research-based program titled “Strategy to Counter Staff Shortage Among Health Professions” was conducted. It focused on the factors influencing the workforce shortage among health professionals and the support of informal caregivers, and included topics such as working conditions (work-related stress, grade-mix and diversity in teams, health promotion), collaboration and competencies (interprofessional, career planning and advanced practice roles, competencies), ethics and security (moral sensitivity, safety culture) and collaboration with informal caregivers [9].

In Switzerland, among health professionals between 2016 and 2018, midwives showed the highest rates of leaving the profession (43.4%), followed by nurses (42.5%), medical-technical professions (41.9%) and medical doctors (31.1%) [10]. A longitudinal study revealed that nurses tend to decide to leave their profession early in their career [11]. Manifold reasons have been discussed with regard to why health professionals are likely to quit their job, including: insufficient preparation for the professional world during education; a theory-practice gap resulting from a disjuncture between ideal practice, taught practice and effective practice in the wards, a lack of fair conflict management [12] and the second victims’ phenomenon [13]. Health professionals may cause unintentionally harm to patients (“first victims”), resulting in a traumatization of the health professionals, as they consider such adverse events as personal failures (“second victim”) [14, 15].

Moreover, health professional students face a double burden owing to the combination of demands from their university (e.g., exams) and from their workplace (e.g., workload), which may preclude them from having a sustainable career in healthcare [16] and potentially lead to long-term consequences such as burnout [17, 18]. Students enrolled in health profession programs have been found to have more lower back and neck pain than their counterparts in other programs [19], and in another study, nursing students reported particularly high levels of stress [20]. During internships or when starting their career, health profession students are confronted with ethical questions. Their decisions may lead to ethical dilemma, if they cannot put their moral judgement into action [21]. An ethical dilemma is the perceived discrepancy between the made moral judgement and the possibility of its realization, which may cause them to experience moral distress [22–24]. Thus, moral distress occurs “when one knows the right thing to do, but institutional constraints make it nearly impossible to pursue the right course of action” [25]. Health professionals today face increasing requirements with regard to their workplace and their employers. Health professionals’ job and workplace must both match with their attitudes, values, ideals, and ethical views [1, 7, 26], and wider institutional and societal objectives risk exacerbating an already challenging situation, leading to ethical dilemmas [27]. Finally, with regard for second victims among health professional students, inadequate support and debriefing was found to increase stress and reduce professional efficacy [28].

Current health profession education curricula have been deemed inadequate in preparing future healthcare workers for the challenges they will face [29]. In a rapidly changing world, it is essential that universities’ curricula stay up to date regarding the necessary competencies for practice along with the implementation of new role models and further changes in the healthcare system, such as ethical education [30]. Maeda and Socha-Dietrich [31] have shown that several competencies, including transversal skills (role crossing skills), communication and collaboration, skills for managing complex tasks, and skills for supporting a positive work culture, are needed to prepare health professionals for diverse challenges. In addition, a lack of attention to, support for and collaboration between health professionals and informal caregivers is often mentioned in national and international literature [32, 33]. This problem is exacerbated during staff shortages, causing dissatisfaction among both informal caregivers and health professionals, who feel they are not doing their job properly.

However, little is known about the extent to which all these important competencies are incorporated and taught in current curricula. Therefore, this study aims to explore the contents of curricula and services for students at universities of applied sciences (UAS) in Switzerland. The knowledge generated may help in identifying gaps in current health profession education curricula and give their developers the possibility to include important educational contents and recommendations to improve not only education, but also the preparedness of various health professionals for their working lives.

## Method

To realize this study’s aim, a qualitative approach called knowledge mapping or mind mapping was used [34, 35]. This method can be compared with content analysis, in which the gathered material is reduced to the core content [34]. The knowledge mapping combines several steps of qualitative interview and analysis technique (data gathering, first content reduction and member check) in the focus interviews and is thus time-saving for the participants and the researchers without loss of data or lack of the necessary depth of data [35]. It was therefore found to come to comparable results with traditional content analysis methods [35].

The method comprises three steps: (a) development of interview guides, (b) conducting the focus group interviews, and (c) analysis and conclusion. The analysis begins during the interviews by visualizing the discussion topics and jointly defining the core topics.

### Recruitment and sample

All 32 directors of the bachelor’s and master’s programs of non-medical health professions (nursing, physiotherapy, occupational therapy, midwifery, nutrition and dietetics, osteopathy, radiologic medical imaging technology, health promotion and prevention, and health sciences) in Swiss UAS, covering the country’s three language regions (German, French, and Italian), were directly contacted via email and asked whether they would be willing to participate in one of five planned focus group interviews (German = 2, French = 2, Italian = 1). Upon agreement, the participants were able to select possible dates among those proposed by the project team. The focus group interviews were conducted with at least three participants each, in a location close to their workplaces.

### Data collection

Data were collected through focus group interviews conducted between October and December 2018. The focus groups were semi-structured using a theory-based interview guide.

### Interview guide

The first step of the knowledge mapping method used was the development of an interview guide. This was developed based on the factors influencing the workforce shortage among health professionals and the support of informal caregivers identified by the “Strategy to Counter Staff Shortage Among Health Professions” project [9]. Experts of the national project formulated questions for their specific research topic, followed by a meeting with all experts to achieve consensus and finalize the semi-structured interview guide (Table 1). The identified factors are the working conditions, collaboration and competencies, ethics and security and the collaboration with informal caregivers as described in the introduction [9]. For example, the topic ‘working conditions’ comprises of the sub-topics ‘work-related stress’, ‘grade mix and diversity’ and ‘health promotion’. Work-related stress is associated with health professionals thinking of leaving the job [7]. This also includes the role conflicts within a diverse team with different grades [7]. Furthermore, inadequate students’ health promotion was found to have negative effects on the student’s health [17, 18]. In addition to the defined topics, an open question was added at the end to capture additional topics from the participants’ perspective. Each topic was given approximately 10 minutes for an even distribution of the time available.

**Table 1:** Semi-structured interview guide

Working conditions	
Work-related stress	What content on work-related stress is taught to students?
	What content on maintaining a work-life balance is taught to students?
	To what extent are students taught about the resulting long-term consequences?
Grade mix and diversity	What content on collaboration among different generations is taught to students?
	What content on collaboration among staff of different grades is taught to students?
	What content is taught to students on intercultural collaboration?
Health promotion	Are there any specific lessons and training focusing on health maintenance and health promotion for students?
**Collaboration and competencies**	
Interprofessional	Is there specific education/training for interprofessional collaboration?
	Is there specific education/training for interprofessional communication?
Career planning and advanced practice roles	To what extent are different professional roles and their respective responsibilities (i.e., research, management, advanced practice roles) addressed?
	To what extent are students taught about career planning?
Political, economic and digital competencies	To what extent are students taught content on how to position themselves politically?
	What content is taught on how to deal with the economization of health professions?
	How are students’ digital competencies promoted?
**Ethics and security**	
Moral sensitivity	What measures are put in place to foster moral sensitivity among healthcare students?
Safety culture	Are aspects of a safety culture (e.g., dealing with mistakes in the team) addressed?
	Is the “second victim” phenomenon (i.e., impacts and traumatization due to undesirable events) being sensitized?
**Collaboration with informal caregivers**	
Informal caregivers	What content on the topic of “informal caregivers” is taught to students?
**What is missing?**	
	What do you think is additionally missing in terms of educational opportunities?

### Preparation of the participants

As thematic preparation for the focus group interviews, all participants received the questions included in the interview guide in English as a PDF via email one month prior to the workshop.

### Focus group interviews

For each language region of Switzerland, one team (comprising a senior researcher and a research assistant) was trained in advance for the second step of the knowledge mapping process, conducting the focus group interviews. As training, a pretest of a focus group interview was conducted within the research group. Each team received an interview guide to ensure that the approaches would be the same across the interviews. The senior researchers all had prior experience in curriculum development. Each workshop was planned to last two hours, including an introduction. Every topic included in the interview guide was discussed. The senior researchers made sure that there was enough time to discuss each topic in the focus group. Given that important additional topics could arise during the interviews, the senior researchers took care to facilitate in-depth discussions of these topics as well. During the interviews, the assistant illustrated one knowledge map per topic on a flip chart, creating a complex network that synthesized the discussion visually. Indeed, according to Vail [36], “[a] knowledge map is the visual display of captured information and relationships.” Central concepts within a topic were arranged in relation to each other, and recurring concepts were highlighted. The knowledge maps also served as records of the focus group interviews. Before moving to a new topic, the senior researcher ensured that all inputs had been documented, and validated the notes with the participants, whether the teams understood the arguments as they have been meant (consensual validation) [34].

### Data analysis

The last step of the method was data analysis and conclusion. Analysis was a multi-stage process. It started during the focus group interviews with the illustration of the knowledge maps by a research assistant. After the interviews, the knowledge maps were digitized using MS Visio®, and all maps from the focus group interviews were merged by topic. Next, the maps were discussed and clustered by two researchers in MS Visio®, resulting in the visualized formation of the relevant clusters. Finally, each cluster was described in the context of the study’s aim by one researcher, and approved by a second researcher.

## Results

**Fig. 1:**
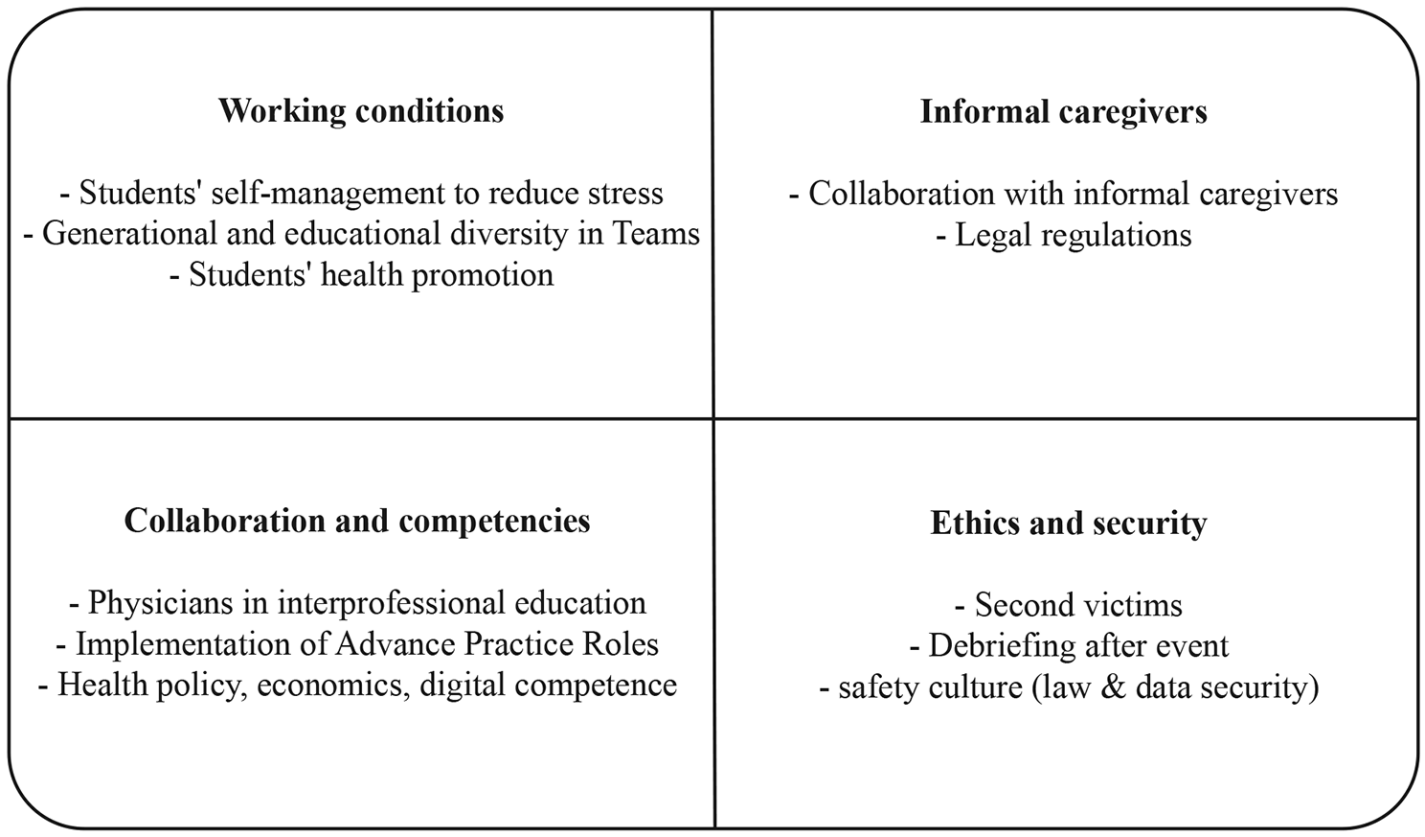
Summary of the identified gaps in current health profession education curricula

Four focus group interviews with 21 directors of bachelor’s and master’s health programs at Swiss UAS were conducted in the German- (n = 2) and French- (n = 2) speaking parts of Switzerland. The participants represented the following professions: nursing, physiotherapy, occupational therapy, nutrition and dietetics, and health sciences. Between three and eight directors of the bachelor’s and master’s programs of non-medical health professions participated per focus group interview (Table 2). Among the programs, the largest number of participants represented nursing (n = 11), followed by health sciences (n = 6), and finally physiotherapy, occupational therapy, midwifery, and nutrition and dietetics (each n = 1). No additional topics were mentioned by the participants besides those defined in the interview guide. In Fig. 1 the identified gaps in current health profession education curricula are summarized. The cluster maps of each topic are available as additional material (Additional file 1–4).

**Table 2:** Sample description

Program	N (%)
Nursing	11 (52)
Physiotherapy	1 (5)
Occupational therapy	1 (5)
Midwifery	1 (5)
Nutrition and dietetics	1 (5)
Health sciences	6 (29)

## Working conditions

### Stress at work

The theoretical concept of stress and its consequences for psychosocial health is taught. Students can profit from internal services, which offer meetings with their supervisors, where they can discuss their stress (e.g., work-life balance, financial situation) and be briefed and debriefed about stress-inducing situations during their internships. During the interviews, the participants implied that through a course on self-management for patients, they also learn about their own self-management. Furthermore, they claimed that lecturers expect a transfer of taught strategies for patients to themselves, although this is neither consciously promoted nor evaluated.

### Grade mix and diversity in teams

Besides nursing and midwifery, other programs generally lack content with regard to collaboration across different levels of qualifications. Moreover, no courses addressing generational and educational differences within teams exist.

### Health promotion

Students are taught in back-friendly work (e.g., kinesthetic) or the importance of healthy nutrition in shift work. Moreover, students offer internal services for other students, such as dietary consultations or massages. Some UAS offer services for their students like access to sport facilities and special courses (e.g., Reiki and mindfulness). None of the master’s programs included in this study provide courses related to occupational health promotion for students. The majority of participants stated that within courses on health promotion for patients, they expect students to adopt similar strategies for themselves.

## Collaboration and competencies

### Interprofessionalism

In some programs, students participate in courses across health professions, although these can differ considerably in their implementation. Thus, whereas in some courses, students from different health professions participate in the same lectures in the same room, in other (generally practical) courses they interact with each other. Furthermore, the participants reported that collaboration with physicians and uniform wording within interprofessional programs are lacking.

### Career planning

The participants stated that they offer internal and external approaches for their students’ career planning. In the internal approach, students can discuss their thoughts concerning their career planning within mentoring session. The external approach comprises a labor market day or workshop, in which employers introduce themselves to future professionals. Furthermore, role models from the practice field present their backgrounds and competencies, giving students the possibility to gain insights into potential future career paths.

### Advanced practice roles

Whereas bachelor’s students only receive brief insights into future potential roles, in the master’s programs advanced practice roles are widely covered. UAS support events for international exchange and are expanding their range of specific courses for advanced practice roles. However, the participants described the missing application of the theoretical models of advanced practice roles taught in practice.

### Political, economic and digital competencies

In the programs, professional associations are invited to share their agendas, debate with students and thereby raise the latter’s awareness of professional policy issues. Nevertheless, the participants stated that there is insufficient input with regard to the mechanisms of health policy and its related consequences for the healthcare system and professionals, such as competencies, wages and rationing.

None of the programs offer courses on economics. However, in programs for professions in which a high proportion of students are expected to be self-employed, this topic is covered to some extent.

Digital competence is developed through some courses, such as practical training with electronic health records and the use of smart devices with patients. Students are expected to possess basic digital competencies, such as the installation of software and the application of writing software, at the start of their program.

## Ethics and security

### “Second victim” phenomenon and safety culture

The participants claimed to be unfamiliar with the “second victim” concept and stated that it is not explicitly discussed in their programs. The participants claimed that students are offered a variety of courses for safety management to minimize the risk of incidents with patients (e.g., medication errors, preventing falls, aggression management), but there is no content on how to debrief after an event. In clinical skills workshops, students are familiarized with safety devices, mobility aids, checklists and incident reports, and they also enact incidents realistically with actors. However, the development of a safety culture also includes topics such as labor law and digital data security. All the participants stated that barely any courses on such topics exist.

### Moral sensitivity

All the participants agreed that they have several courses on ethics in practice as well as in research. Students can learn about the history of ethics, discuss dilemmas, and practice how to consult patients in filling out a patient decree. In the master’s programs, students learn how to write an ethics proposal for research projects and are taught about the underlying laws such as the Human Research Act. Furthermore, ethical decision-making skills are core competencies of the advanced practice roles taught in the master’s programs.

## Collaboration with informal caregivers

The vast majority of the programs’ directors mentioned not having any specific course related to collaboration with informal caregivers and their need for support. Moreover, theoretical input with regard to the underlying legal regulations (such as the child and adult protection authorities, and national or local services for the financial support of informal caregivers) is not considered for courses. In some programs, students learn how to collaborate and assist informal caregivers to enhance support for relatives in need, such as by focusing on the former’s expertise, needs assessment and education.

## Discussion

This study has provided knowledge regarding the contents and services of the bachelor’s and master’s programs for non-medical health professions at Swiss UAS. The findings highlight how curricula need to improve to meet the requirements for future health professionals’ competencies. According to our focus group interviews, UAS’ curricula are heterogeneous with regard to the predefined topics they cover. For example, only one participating UAS proved effective in integrating informal caregivers into its curricula. These differences may indicate that the contents of curricula depend on the research interests and expertise of lecturers and not on future needs. The findings also reveal overlaps across topics in terms of missing or available measures, indicating clusters for suitable measures. The most important statements of recommendations based on the study findings are used as section headings: preparatory programs for coping strategies, new and advanced competencies, interprofessional collaboration and diversity, support services for students, and collaboration with informal caregivers.

### Preparatory programs to cope with stressful working conditions

Several topics are included in Swiss UAS’ current curricula, but with a focus on patients’ and not on students’ needs (e.g., self-management and health promotion in stressful situations). Coping strategies for managing work-related stress or moral distress are underrepresented, which could lead to negative strategies and hamper the learning process [23]. The insufficient preparation of health profession students for the stress factors they are expected to face after graduation, such as a lack of self-care and stress management skills education in preparatory programs [20], and the provision of student self-awareness skills [37], has also been discussed internationally. However, courses to improve self-care techniques throughout the programs on offer should prove beneficial. In particular, studies on mindfulness-based interventions have concluded that such practice can effectively reduce stress at work among health profession students [38–40]. Nonetheless, the length of programs to enhance mindfulness has been shown to be crucial for achieving the expected stress-reducing effect [41].

### New and advanced competencies

New and advanced competencies to manage digital technologies or to argue in favor of oneself and one’s patient in the context of economy and ethics, do not seem to be sufficiently addressed to meet requirements. These findings are in line with discussions regarding the development of professionalism in healthcare, which emphasize that in addition to medical knowledge and skills, health profession students should dedicate themselves to political, economic and social questions [42, 43]. This is important because health professionals should understand money flows and their role in such dynamics, as well as participate in political discourse to represent and strengthen their own profession [44]. Nevertheless, there seems to be a consensus on medical core competencies, such as evidence-based practice, at least from a scientific perspective [45]. However, according to this study’s findings, it remains unclear exactly what competencies students of the future need in the context of digitalization. Educational organizations are expected to prepare students for the future so that they can become productive employees. Thus, they should at least integrate digital technologies into their courses, which students already use in their private lives and practice, such as simulations (whether augmented or virtual), electronic health records, and smartphone technology [46].

### Interprofessional collaboration and diversity in teams

For a long time now, the model whereby students from different disciplines are taught in the same room is not understood as interprofessional [47]. Therefore, the results of this study show the obsolete educational implementation of interprofessional collaboration in UAS’ programs, although admittedly the implementation of interprofessional collaboration in some programs was still being developed. However, this does not seem to be an issue unique to Switzerland, and propositions have been internationally discussed to proceed in successful interprofessional education [48, 49]. Furthermore, the results reveal a lack of involvement of physicians in the education of non-medical professions. This difficulty is underlined by the predominant stereotypes of the different professions [50], which must be overcome in advance by focusing on attitudes and perceptions toward interprofessional collaboration already in education [51].

In addition to interprofessional collaboration, the findings indicate that insufficient attention is currently paid to diversity in teams, such as their composition with regard to culture and age. This is especially important, because in many countries numerous health professionals in the workforce are migrant workers or immigrants, who were generally educated in their home countries [52]. For example in Switzerland, 33% of health professionals are migrant workers [53]. Their education and professional values may be very different from those of their counterparts educated in Switzerland. This may lead to misunderstandings or conflicts about how professional situations are handled. Therefore, it seems necessary to adapt curricula to sensitize students to the cultural composition of teams in healthcare, as well as culture’s effects on team dynamics and the handling of the people receiving care. In particular, curricula should operate to eradicate conscious and subconscious prejudicial and stereotypical thinking about racial and ethnic minority groups [54–56].

### Services for students

Among the UAS included in this study, not all claimed to have services for briefing and debriefing before and after an internship, or to promote services to support students’ health. Social support, like informal or formal exchange with colleagues or mentors, is crucial to adopt sustainable coping skills [57, 58]. Educational institutions should offer services for formal exchange and provide infrastructure and time for informal exchange. With regard to physical activities, more engagement to promote the services on offer is expected to be beneficial, as the availability of a service does not immediately lead to its beneficial use [59].

### Collaboration with informal caregivers

Through the interviews it became apparent that depending on the thematic focus of an organization and its associated health profession, informal caregivers’ involvement differs considerably. The participating UAS should exchange knowledge among each other with regard to their experiences of collaborating with informal caregivers in education. To do so, however, the UAS should be aware of their current level of involving informal caregivers, which can be achieved with the help of Rhodes [60] ladder of involvement. For the healthcare system and for health professionals, informal caregivers play an important role, hence insufficient support and collaboration results in negative effects on their health [32, 61]. By integrating them as partners into health professionals’ education, greater long-term interaction between these two groups can be promoted and ensured [60].

### Strengths and limitations

This study aimed to focus on all three of Switzerland’s language regions and include all of its UAS. Ultimately, it achieved a participation rate of 65% of the relevant study population. No interview in the Italian-speaking part of the country was conducted, limiting the results to an overview of the German- and French-speaking parts. Nonetheless, the latter two make up the majority of Switzerland’s population. One strength of focus group interviews is their ability to stimulate discussion among a group of people with similar experiences or expertise [62]. Furthermore, member checking was conducted with the participants during the interviews. The first step of the analysis required the participants to validate the written topics, that is, whether or not they agreed with the statements provided. The participants could also clarify potential misinterpretations and give their consent to the conclusions drawn. Furthermore, the moderators were experienced researchers, having worked for several years on relevant topics in the field of higher education. This was important because it enabled the participants to feel comfortable that the moderators understood topic-specific terms. Relatedly, the moderators ensured that the participants had equal time to speak [63].

One limitation of the study was that no differentiation between the bachelor’s and master’s programs of the non-medical health professions was possible because of the recruitment process and the composition of the focus groups. It was not possible to organize focus groups by profession because there were insufficient numbers of participants in some. A further limitation was that by predefining the topics for the interviews via a guide, other relevant topics were less likely to emerge. Consequently, it is possible that some important aspects (such as management and leadership) remained untouched and so should be explored and discussed in the future. However, the participants did not mention any additional topics.

Furthermore, using the knowledge mapping method, no quotes from the participants were recorded, since the knowledge maps served as records of the interviews. However, all participants validated each map before each change of topic during the focus groups. Nonetheless, quotes from participants would have increased the traceability of the analysis process and illustrated the condensed findings [64].

## Conclusion

The contents of health profession curricula in Swiss UAS are heterogeneous but consistently fail to develop important competencies with regard to future challenges and actual high demands in the COVID-19 pandemic to maintain health in working life. This urges for a national exchange to harmonize contents and to learn from each other on how to educate and prepare health professionals for future challenges and workforce retention. This study has helped fill the gap regarding what topics should be integrated in health profession curricula to meet contemporary needs and to prepare them for the challenges they will face in their daily practice in the future. Advanced services for students besides the content of lectures, such as supervision in transferring theory into practice and debriefing after an internship, may help improve their practical competencies. Additionally, students should learn coping strategies concerning how to reduce personal stressors and manage work-related stress or moral distress at work. As indicated in the Discussion, previous studies on mindfulness-based interventions have highlighted promising improvements in stress management.

In the context of increasingly rapid changes through, for instance, innovations in technology, programs should prospectively include students as well as representatives from practice to continuously evaluate the relevance of the contents of their curricula. With regard to investments aimed at improving interprofessional education, relevant international frameworks could provide a solid basis. To increase informal caregivers’ involvement and collaboration, educational organizations should exchange their knowledge and experiences to learn from other educational organizations, which are advanced in this process with promising achievements. This study’s findings help sensitize directors of bachelor’s and master’s programs for the topic, and raise questions that should be answered in future research, such as with regard to the development of skills that can promote successful collaboration with informal caregivers, and how best to sustainably protect the health of healthcare professionals and informal caregivers. Switzerland is not the only country affected by a workforce shortage among health professionals: this issue has been described across a multitude of countries. Therefore, a lack of preparation to face the stressors experienced by healthcare professionals in the healthcare labor market does not seem to be country-specific. Hence, the contents of curricula need to be evaluated with regard to this gap. It can be assumed that different priorities will be defined based on the results.

## Electronic supplementary material

Below is the link to the electronic supplementary material.

Supplementary Material 1

Supplementary Material 2

Supplementary Material 3

Supplementary Material 4

## Data Availability

The datasets generated and/or analysed during the current study are not publicly available as the knowledge maps generated as datasets allow conclusions to be drawn about the participants but are available from the corresponding author on reasonable request.
