# Supplementary figures and images for: Preparing students to deal with the consequences of the workforce shortage among health professionals: a qualitative approach
Source: BMC Med Educ. 2022 Nov 4;22:756. doi: 10.1186/s12909-022-03819-4 (PMC9636659; doi:10.1186/s12909-022-03819-4)

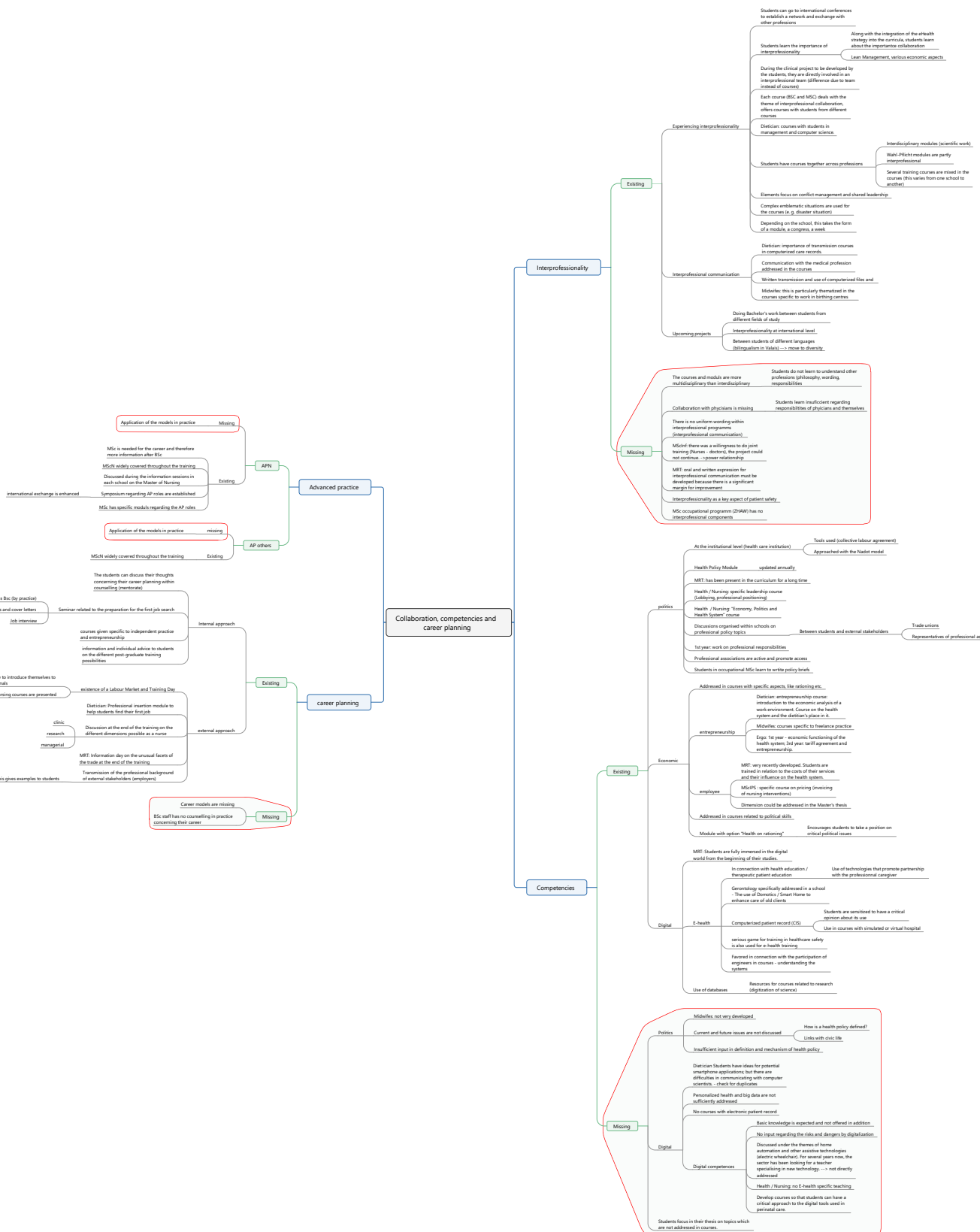

Supplement: Supplementary file 1 — Supplementary Material 1 [file 12909_2022_3819_MOESM1_ESM.pdf]

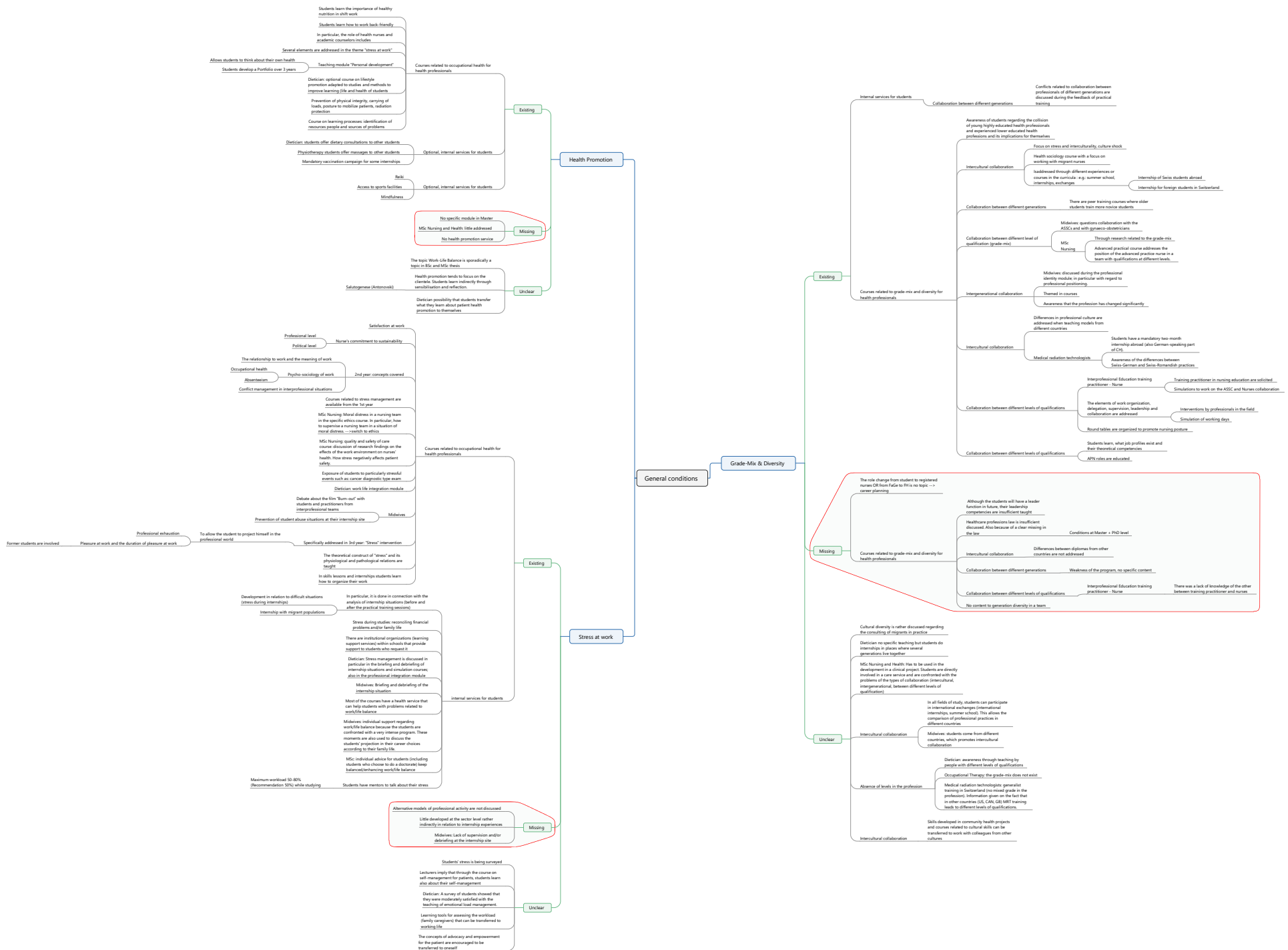

Supplement: Supplementary file 2 — Supplementary Material 2 [file 12909_2022_3819_MOESM2_ESM.pdf]

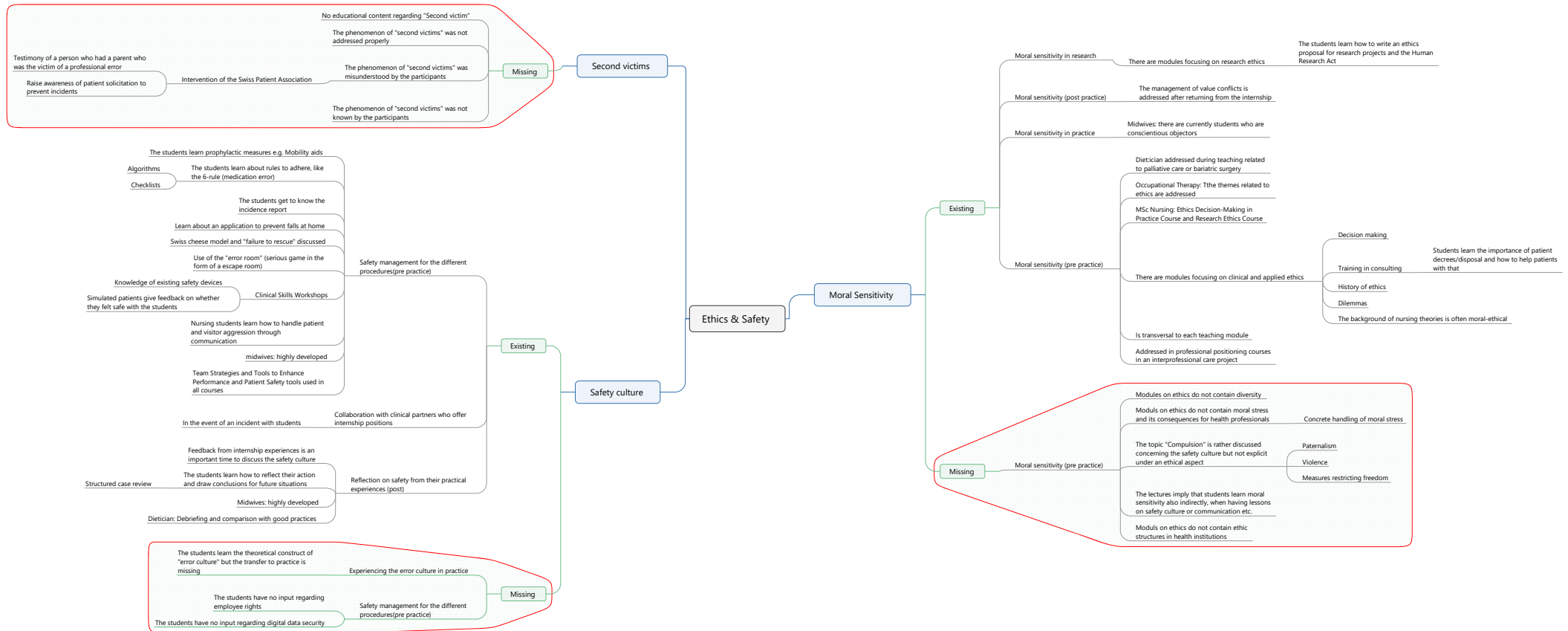

Additional file 3: Cluster map ethics safety

Supplement: Supplementary file 3 — Supplementary Material 3 [file 12909_2022_3819_MOESM3_ESM.pdf]

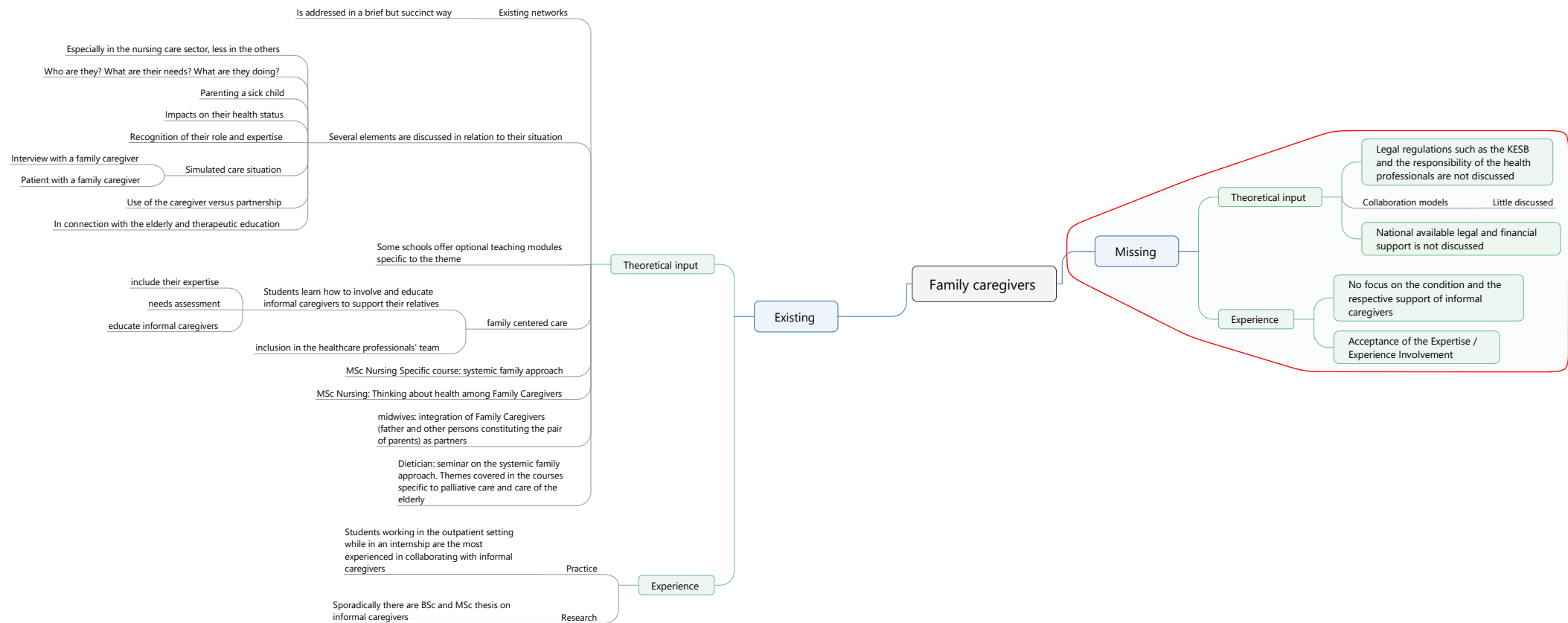

Additional file 4: Cluster map informal caregivers

Supplement: Supplementary file 4 — Supplementary Material 4 [file 12909_2022_3819_MOESM4_ESM.pdf]
